# Morphological Brain Age Prediction using Multi-View Brain Networks Derived from Cortical Morphology in Healthy and Disordered Participants

**DOI:** 10.1038/s41598-019-46145-4

**Published:** 2019-07-04

**Authors:** Joshua Corps, Islem Rekik

**Affiliations:** 1 BASIRA lab, School of Science and Engineering, Computing, University of Dundee, Dundee, UK; 2 Faculty of Computer and Informatics, Istanbul Technical University, Istanbul, Turkey

**Keywords:** Machine learning, Cognitive ageing

## Abstract

Brain development and aging are dynamic processes that unfold over years on multiple levels in both healthy and disordered individuals. Recent studies have revealed a disparity between the chronological brain age and the ‘data-driven’ brain age using functional MRI (fMRI) and diffusion MRI (dMRI). Particularly, predicting the ‘brain age’ from connectomic data might help identify relevant connectional biomarkers of neurological disorders that emerge early or late in the lifespan. While prior brain-age prediction studies have relied exclusively on either structural or functional connectomic data, here we unprecedentedly propose to predict the *morphological age of the brain* by solely using *morphological brain networks* (derived from T1-weighted images) in both healthy and disordered populations. Besides, although T1-weighted MRI was widely used for brain age prediction, it was leveraged from an image-based analysis perspective not from a connectomic perspective. Our method includes the following steps: (i) building multi-view morphological brain networks (M-MBN), (ii) feature extraction and selection, (iii) training a machine-learning regression model to predict age from M-MBN data, and (iv) utilizing our model to identify connectional brain features related to age in both autistic and healthy populations. We demonstrate that our method significantly outperforms existing approaches and discovered brain connectional morphological features that fingerprint the age of brain cortical morphology in both autistic and healthy individuals. In particular, we discovered that the connectional cortical thickness best predicts the morphological age of the autistic brain.

## Introduction

The development and aging of the human body are complex processes. In particular, individual development and aging in both healthy and disordered participants progressively alter the morphology of the brain. There is an ample literature investigating how brain structures change with aging including volume loss^1–5^ and reduction in white matter integrity^6^ in healthy individuals. Unique aging patterns have been associated with different brain disorders such as dementia, where specific brain regions undergo an accelerated aging process, implying accelerated brain atropy^1,7^ More recent studies^8–11^ on brain development have shown that neurological disorders such as Autism Spectrum Disorder (ASD) and Alzheimer’s Disease (AD) can cause a disparity between the chronological brain age of a subject, that is the number of years since birth, and a ‘data-driven’ brain age, or how old they seem to be, which can be quantified by neuroimaging data, also known as their ‘biological age’ or ‘physiological age’. This disparity between chronological and biological age in subjects with a neurological disorder, is often the result of accelerated or decelerated aging of their brain caused by the disorder. By using a calculated data-driven brain age and its deviation from the chronological age, a prediction can be made on whether an individual has a disordered or healthy brain. Additionally, many neurological disorders, such as ASD, currently do not have widely accepted biomarkers^5^ for the disorder, and so by predicting the brain age and calculating delayed or accelerated age, it may be possible to link these features to the disorder.

The majority of studies in recent years for predicting biological age from connectomic brain data tend to focus on the use of MRI (Magnetic Resonance Imaging) such as functional MRI (fMRI)^12,13^ or diffusion MRI (dMRI)^13,14^. However, both of these techniques have limitations which can restrict their usefulness for this task. Firstly, fMRI data can be very noisy. Additionally, the high variability in dMRI tractography methods^15^ can introduce a bias into the data, somewhat skewing the results of any analysis. On the other hand, there is little research on biological age prediction using morphological brain networks, despite the fact that recent research^16,17^ has highlighted that there may be a link between morphological features, such as cortical thickness and sulcal depth, and different neurological disorders, such as ASD. Previously, Brown *et al*.^14^, utilized diffusion MRI data to predict the biological age of preterm infants and then used this to calculate what they called the ‘Relative Brain Network Maturation Index’ (RBNMI), which is defined as the absolute value of the predicted age of the infant minus their true age. The results of their research showed that the development of structural connectomes in preterm neonates with abnormal, disordered, development was delayed. Shen *et al*.^18^, recently proposed a new state-of-the-art method for predicting behavioural scores from fMRI data called Connectome-Based Predictive Modelling (CPM). CPM works by extracting and summarizing the most relevant features from connectomic data into positive or negative features. These features are used to train a machine-learning regression model to predict behavioural scores for new subjects. In their evaluation, the model was limited to testing with functional MRI data, however, it can be applied to other types of brain imaging data.

Additionally, recent works on neurological disorder diagnosis using morphological brain networks^19–23^ derived from T1-w MRI have developed a new research direction unifying brain connectomics and morphology. Although several works have largely utilized T1-w MRI for estimating the brain age of healthy subjects in children and adolescents^24,25^ as well as elderly subjects with neurodegenerative disorders^7,26^, these have only investigated low-order morphological measurements such as volume or image intensity. This overlooks the high-order complex relationship between brain regions, which work as an interconnected system from early development till aging. Hence, investigating brain age on a high-order connectional level using a network representation derived from T1-w MRI is overlooked. Particularly, to the best of our knowledge, using morphological brain networks on a regression problem, such as predicting age or cognitive scores, remains unexplored. To fill in this gap, we unprecedently propose to predict *‘the morphological age of the brain’* by using multi-view morphological brain networks (M-MBNs), each view quantifying a specific trait of the cortex morphology (e.g., curvature). Then, the most relevant features are selected from the morphological age prediction task, which identifies the morphological brain features that best predict the chronological brain age. An ensemble machine learning regression model is then built to produce multiple biological age predictions, then combined to give the final age prediction. Finally, the most selected features by our model are used to identify features that most correlate with the morphological brain age (MBA) and how a neurological disorder, such as ASD, can affect those features. The identified brain features can potentially reveal biomarkers of the target disorder.

Finally, while there is extensive work on the topic of using machine-learning and brain neuroimaging data for classifying disease types^27–29^, the majority of these studies approach the problem from the same perspective, simply using the connectomic data to classify a subject. We will attempt to approach this from a new angle, instead of using the connectomic data to directly classify a disease state, we will instead utilize the MBA disparity that we will calculate by predicting our ‘data-drive’ brain age, combined with the morphological connectomic data, to boost classification accuracy in neurological disorders.

We compare our proposed framework against multiple benchmark methods including the current state-of-the-art CPM^18^ framework. More importantly, using our framework, we investigate the relationship between brain connectional morphology and brain development and neurological disorders in two folds: (1) identifying the morphological connectional features that are most correlated with the MBA, and (2) classifying healthy and disordered brain states using the disparity between predicted morphological age and the true age.

## Results

### Dataset and parameters

To evaluate our proposed method, we used 5-fold cross-validation on two different populations from the ABIDE data (http://fcon_1000.projects.nitrc.org/indi/abide/) (Supplementary Table 1): 186 of which were Normal Control (NC) subjects, with a mean age of 16.65 ± 6.06, and 155 Autism Spectrum Disorder (ASD) subjects, with a mean age of 16.92 ± 6.38. The data for each subject was composed of a T1-w MRI scan. We used FreeSurfer^30^ to reconstruct both the right and left cortical hemispheres (LH and RH) for each subject from their T1-w MRI scan. Using Desikan-Killiany Cortical Atlas, each cortical hemisphere was then parcellated into 35 cortical regions. Next, we constructed the multi-view morphological brain network (M-MBN), composed of 4 views, each derived from a specific cortical measurement: 1) the maximum principal curvature, (2) the mean cortical thickness, (3) the mean sulcal depth, and (4) the mean average curvature.

### Comparison methods

We compared our proposed framework against multiple benchmark methods. The first is the state-of-the-art framework, Connectome-Based Predictive Modeling^18^ (CPM). We also compared against Support Vector Regression (SVR), SVR with Recursive Feature Elimination (RFE) for feature selection, and Random Forest (RF). For these comparisons, we benchmarked each method using the 4 individual MBN views, as well as our combined averaged (AVG) and concatenated (CON) MBN, except for in the case of CPM where it cannot use the CON MBN due to the input data being limited to an *R* × *R* matrix of features, where R denotes the number of regions of interest (ROIs). Each comparison method was evaluated using 5-fold cross-validation (CV).

### Regression random forest parameters

The range of selected features and increment varied based on the data structure used, in the case of single views and averaged views the number of features selected ranged from 50 to the maximum and increased in increments of 50. For concatenated views, the selected features ranged from 100 to maximum. For the regression random forest, we varied the number of trees from 20 to 200 with an increment of 20 per iteration, and ultimately set to 150 trees. We empirically set the ‘minimum leaf size’ to 5 and set the ‘number of predictors to sample’ to one third of the number of variables.

### Evaluation results

**Figure 1:**
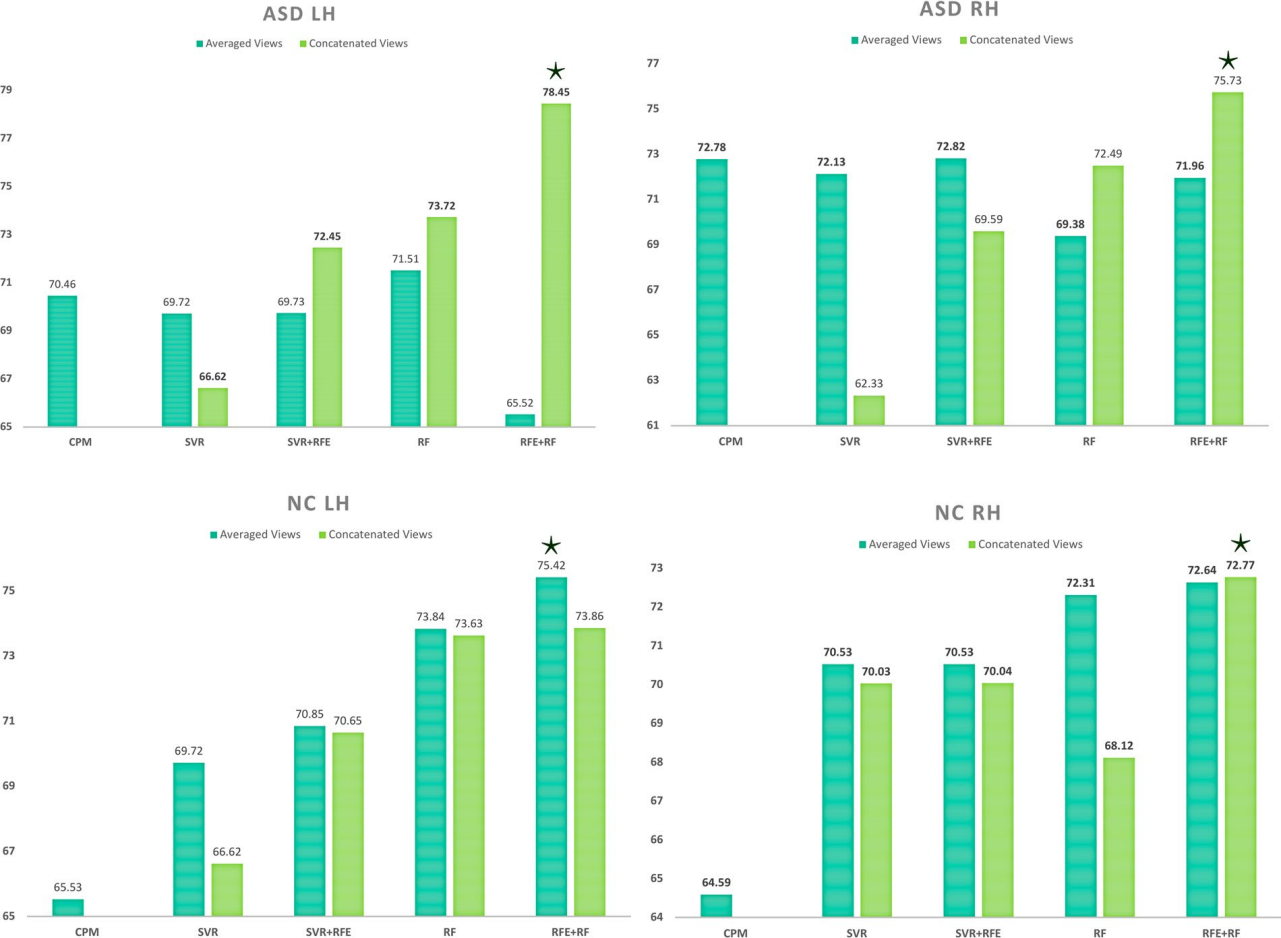
Age prediction accuracy for each of the 4 datasets (ASD Left Hemisphere, ASD Right Hemisphere, NC Left Hemisphere, and NC Right Hemisphere) using 4 benchmark methods against our proposed method (far right). Each method was evaluated on both averaged views and concatenated views.

Tables 1 and 2 report the MBA prediction results for ASD LH and NC LH, respectively. Supplementary Tables 2 and 3 report the MBA prediction results for ASD RH and NC RH, respectively. As can be seen in these tables, when using single views, view 2 (i.e., cortical thickness) performs substantially better than the other views. Figure 1 displays the results for only concatenated (CON) and averaged (AVG) views by all methods. Overall, the best accuracy was achieved using CON views. As shown in Fig. 1 our method achieved the highest age prediction accuracy using both Pearson correlation and MAE as evaluation criteria with an improvement of ~3–5% for ASD data and ~2–3% for NC data.

**Table 1:** Comparison of age prediction using **ASD LH** data with 5-fold cross-validation by our method and comparison methods.

Method	Dataset	R	P	MAE
CPM	View 1	0.43	1.61E-08	4.31
	View 2	0.68	9.90E-23	3.57
	View 3	0.56	5.00E-14	3.92
	View 4	0.43	2.40E-08	4.46
	Averaged Views	0.70	1.48E-24	3.39
SVR	View 1	0.45	3.55E-09	4.10
	View 2	0.70	2.29E-24	3.46
	View 3	0.48	1.94E-10	4.09
	View 4	0.06	4.34E-01	4.65
	Averaged Views	0.70	7.00E-24	3.38
	Concatenated Views	0.67	3.07E-21	3.72
SVR + RFE	View 1	0.46	2.41E-09	4.10
	View 2	0.71	4.78E-25	3.41
	View 3	0.56	2.95E-14	3.80
	View 4	0.06	4.34E-01	4.65
	Averaged Views	0.70	6.91E-24	3.38
	Concatenated Views	0.72	1.65E-26	3.29
RF	View 1	0.54	4.12E-13	4.01
	View 2	0.73	7.62E-27	3.56
	View 3	0.54	5.38E-13	3.97
	View 4	0.43	3.02E-08	4.48
	Averaged Views	0.72	1.43E-25	3.53
	Concatenated Views	0.74	7.76E-28	3.45
RF + RFE (ours)	View 1	0.56	2.09E-14	3.92
	View 2	0.75	9.51E-29	3.49
	View 3	0.52	4.48E-12	3.96
	View 4	0.33	2.24E-05	4.48
	Averaged Views	0.66	2.27E-20	3.73
	Concatenated Views	**0.78**	**1.47E-33**	**3.21**

R is the correlation between the predicted ages and the ground truth ages, and P is the p-value of their statistical difference. MAE denotes the mean absolute error between the predicted ages and the ground truth ages.

**Table 2:** Comparison of age prediction using **NC LH** data with 5-fold cross-validation by our method and comparison methods.

Method	Dataset	R	P	MAE
CPM	View 1	0.45	1.08E-10	4.05
	View 2	0.67	2.02E-25	3.32
	View 3	0.37	2.72E-07	4.25
	View 4	0.39	3.66E-08	4.29
	Averaged Views	0.66	3.42E-24	3.40
SVR	View 1	0.45	3.55E-09	4.10
	View 2	0.70	2.29E-24	3.46
	View 3	0.48	1.94E-10	4.09
	View 4	0.06	4.34E-01	4.65
	Averaged Views	0.70	7.00E-24	3.38
	Concatenated Views	0.67	3.07E-21	3.72
SVR + RFE	View 1	0.28	1.17E-04	4.12
	View 2	0.69	1.78E-27	3.23
	View 3	0.42	2.56E-09	3.97
	View 4	0.15	4.74E-02	4.33
	Averaged Views	0.71	1.16E-29	3.10
	Concatenated Views	0.71	1.96E-29	3.09
RF	View 1	0.53	1.24E-14	3.84
	View 2	0.73	8.08E-32	3.11
	View 3	0.35	7.28E-07	4.14
	View 4	0.43	7.21E-10	4.14
	Averaged Views	0.74	8.10E-31	3.05
	Concatenated Views	0.74	4.82E-33	3.17
RF + RFE (ours)	View 1	0.53	4.05E-15	3.82
	View 2	0.73	1.90E-32	3.08
	View 3	0.36	3.96E-07	4.14
	View 4	0.44	3.81E-10	4.11
	Averaged Views	**0.75**	**1.88E-35**	**2.92**
	Concatenated Views	0.74	2.48E-33	3.03

R is the correlation between the predicted ages and the ground truth ages, and P is the p-value of their statistical difference. MAE denotes the mean absolute error between the predicted ages and the ground truth ages.

### Morphological brain connections fingerprinting the biological brain age

**Figure 2:**
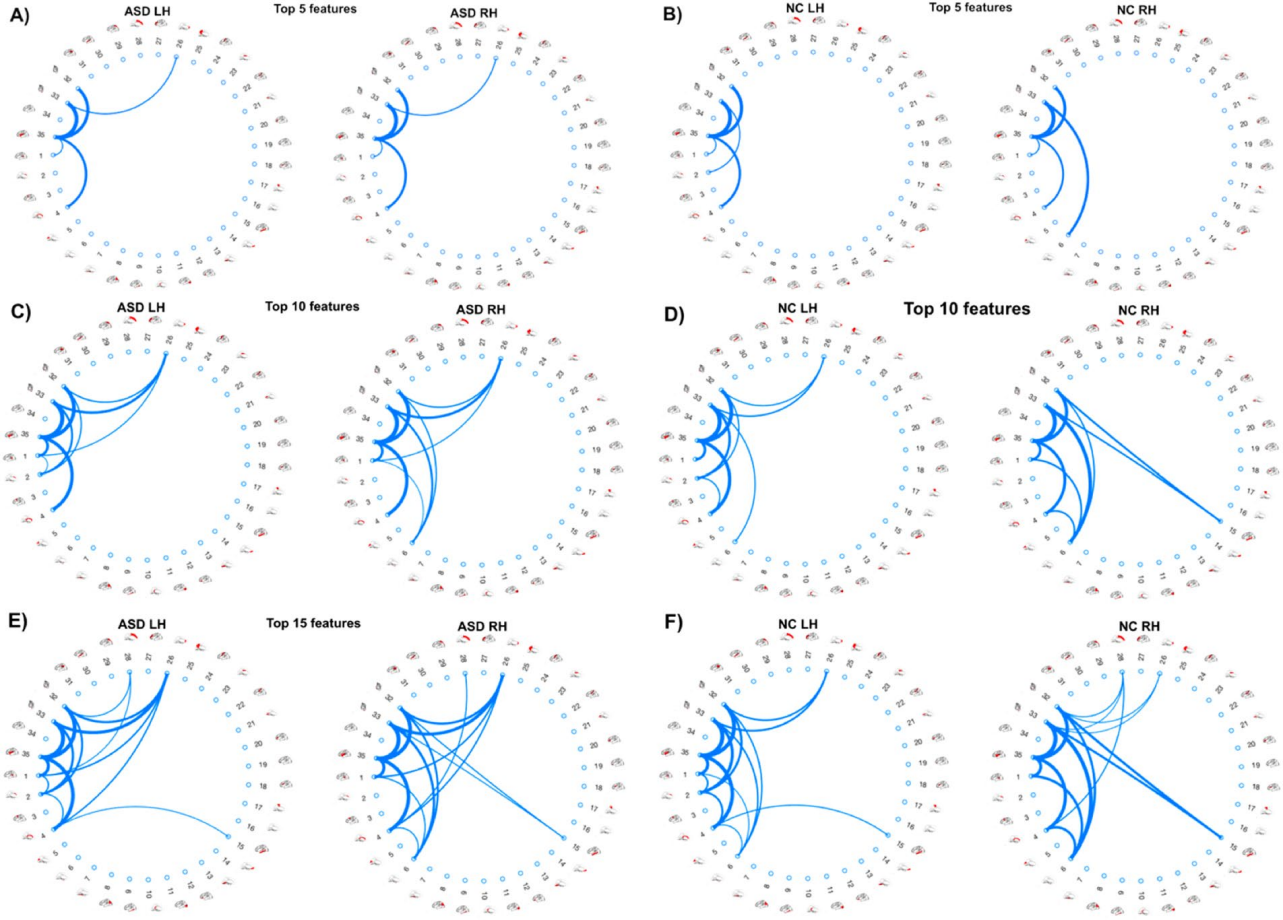
*Identification of morphological connectional features fingerprinting brain age*. Circular graphs showing the top ranked 5 (**A**,**B**), 10 (**C**,**D**), and 15 (**E**,**F**) morphological connectional features that correlate most with age when using concatenation to combine the morphological views. Thicker edges indicate higher correlation with brain age.

**Figure 3:**
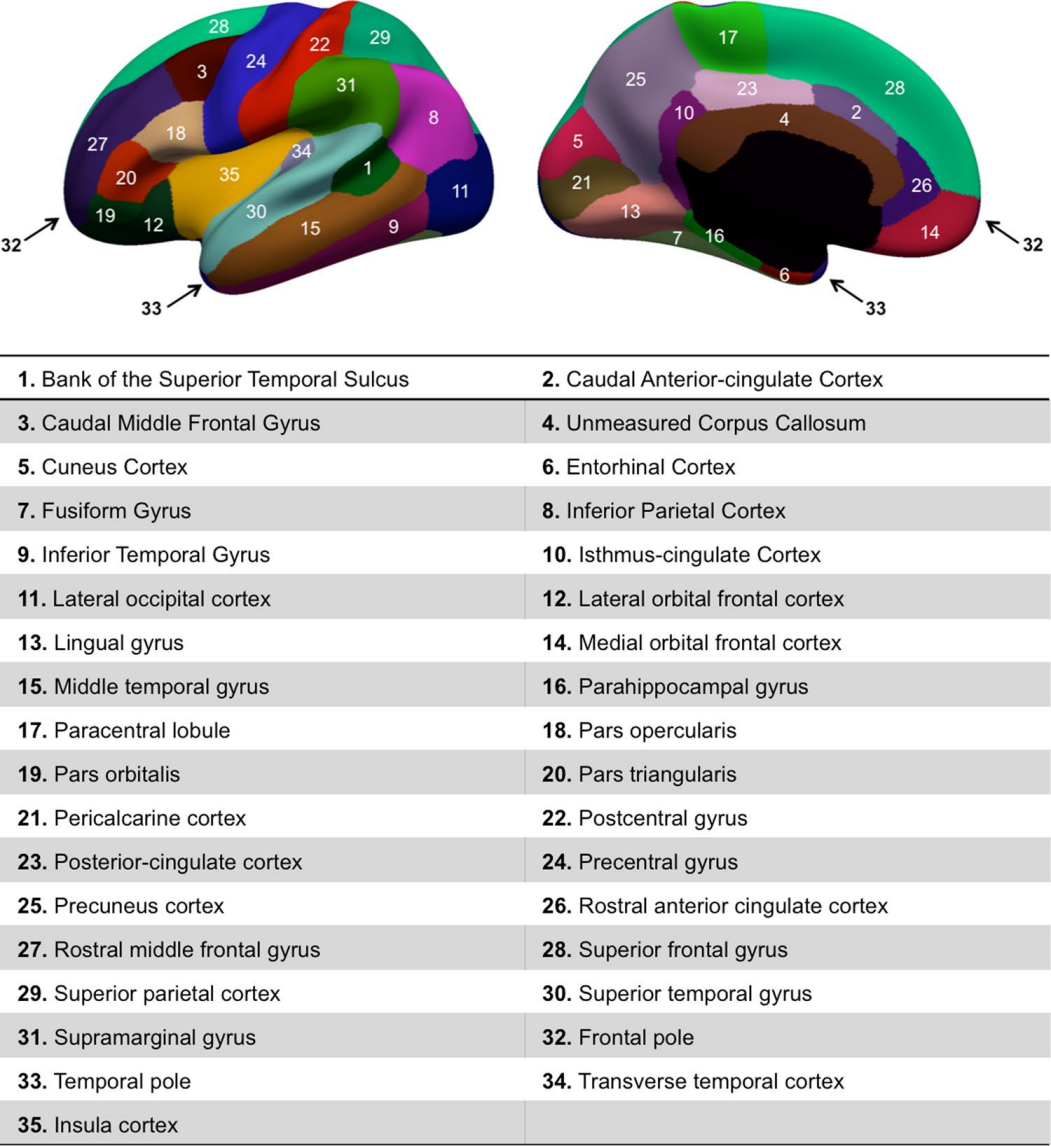
Cortical brain regions of interest used for morphological brain network reconstruction. The numbers with corresponding names can be linked to the circular graphs in Fig. 2.

We also evaluated our method for the discovery and identification of morphological connectional features that are most correlated with the MBA. We identify the top *K* features, in this case we selected the top 5, 10, and 15 ranked features and visualized their connections using circular graphs. Since our aim was to find the most discriminative features, we utilized CON for combining the multiple views instead of AVG. From this, we noted that when using CON, the majority of top highly ranked features were selected from view 2 (i.e., cortical thickness) when selecting a smaller number of features. Due to this, for all hemispheres and datasets (ASD LH, ASD RH, NC LH, NC RH) we identified view 2 as the view with the highest discriminative power and as such the top connections we visualized in Fig. 2 are all from this view. Figure 3 shows the brain regions referred to in the circular graphs. This allows us to identify potentially which regions of the brain and which measurements correlate most with MBA, as well as potentially identifying the different highly correlated features between the healthy (NC) and disordered (ASD) subjects and how ASD manifests itself in these connections. The thickness of each edge connecting two ROIs represents the rank of the connection as given by RFE in the feature selection step. The higher ranked, more discriminative, a feature is then the thicker the connection between the ROIs and conversely, the lower ranked, less discriminative, a feature is a thinner connection line is then used.

As revealed in Fig. 2 the most discriminative morphological connectional features for NC LH connected the Insula Cortex (IC) (Region 35) and the Temporal Pole (TP) (Region 33), IC and Frontal Pole (FP) (Region 32), IC and Unmeasured Corpus Callosum (UCC) (Region 4), TP and Rostral Anterior Cingulate Cortex (RACC) (Region 26), and IC and Bank of the Superior Temporal Sulcus (BSTS) (Region 1). Interestingly for the RH, the rankings of the top 5 discriminative features were the same as with the LH on the NC subjects. As for ASD, with the LH the most discriminative morphological connectional features connected IC and TP, IC and FP, IC and UCC, TP and Caudal Anterior-cingulate Cortex (CAC) (Region 2), and TP and RACC. For the RH, the most discriminative morphological connectional features connected IC and TP, IC and FP, TP and (Region 6), IC and UCC, and TP and RACC. With only 5 of the top features selected we note mostly the same connections visualized across different datasets. However, with more selected connectional features correlating best with the MBA, a discriminative pattern emerges between healthy and disordered brains (e.g., connection between posterior cingulate cortex and temporal/frontal poles) and can begin to identify hubs for many different connections.

## Discussion

We presented the seed work on predicting the morphological brain age using multi-view morphological brain networks in healthy and autistic populations. Although our method is simple, our findings are unprecedented and give insights into how the brain age is encoded in multi-view morphological brain networks derived from solely T1-w MRI. Existing studies aiming to predict the brain age have so far relied on functional or diffusion MRI. Our design arose from a need to use T1-w data for investigating the brain morphological connectivity using MRI sequences conventionally acquired in clinical hospitals.

Our proposed framework substantially outperformed the comparison methods when using our multi-view networks. For ASD, the best prediction results were produced by CON in both left and right hemispheres. For NC, in the LH CON marginally outperformed AVG and in the RH AVG performed the best. When using ASD data, the difference between CON and AVG was more substantial. This could suggest that ASD affects morphological features in ways that can only be captured with the complementary information that concatenation retains over averaging. We also noted that the 2^nd^ view (i.e., cortical thickness) achieved significantly higher accuracy than the other individual views (Table 1 and 2, Supplementary Tables 2, 3). Additionally, the top 5, 10, and 15 connections shown in Fig. 2 were all derived from the 2^nd^ view. Our finding is in line with the brain age literature, where reduction in cortical thickness was linked to age in elderly populations with healthy brains^31,32^. Additionally, studies^17,33^ have shown an increase in cortical thickness in subjects with ASD. Our finding suggests that the link between cortical thickness-derived morphological connections and brain development can be applied to more than just elderly populations. More importantly, this might indicate that ‘*connectional cortical thickness’* fingerprints the morphological age of the brain. This is supported by our findings that *cortical thickness* is a key identifier of age, with cortical thickness increasing during early development and decreasing during later aging^21^. Due to this, younger ASD subjects show as being older due to the increased cortical thickness caused by ASD which is supported by Hardan *et al*.^33^, where ASD subjects had an increase in cortical thickness over NC subjects. With older ASD subjects, due to the originally increased cortical thickness, the decrease in cortical thickness caused by normal aging is less noticeable and so identifies the subject as being younger. A more recent study^22^ showed that ASD affects the morphological structure of the cortex in both right and left hemispheres on different connectional levels. In support of our findings, the cortical thickness was identified as the most discriminative feature of the autistic cortex, particularly in the left hemisphere.

From our circular graphs, Fig. 2, we identified the most discriminative features for brain age. We noticed many connections involving the Insular Cortex (IC) in both Healthy and ASD subjects making this a central hub. This agrees with previous findings^34^, which identified relationships between age and emotional development and decision-making and linked these to changes in age and IC. Additionally, we also noticed hubs that appeared more prominent in ASD when compared with NC subjects. One of these hubs is the Rostral Anterior Cingulate Cortex (RACC) which we identified as showing more connections in ASD subjects with the connections also appearing higher in the ranking (Fig. 2). This links to previous research^35^, which identified that ASD subjects showed increased activation of the RACC over Healthy Control (HC) participants in their study. These differences in hubs of connections between ASD and NC subjects may potentially identify biomarkers of the disorder.

As previously shown, our method greatly outperforms the tested state-of-the-art method CPM^18^ for all datasets, which suggests that our method can also be used to predict other clinical scores such as behavioural or cognitive scores in the future and is not just limited to brain age prediction. One limitation of our method is that we use simple methods for combining the data multiple views. To overcome this, in our future work we could investigate more complex methods of combining the views. Additionally, the overall method can be considered as quite simple. This can be perceived as both a limitation, in that there may be potential to get improved results using more complex methods and in the future, this is a research direction that can be investigated. Furthermore, we can also use a-priori feature selection based on biological knowledge to further boost our MBN-based predictive model. However, our original goal was to avoid extremely time-consuming methods for potential use with near real-time results in a clinical setting. Finally, we only utilised data from a single time point. It may be interesting to investigate how longitudinal brain development influences the predicted MBA.

## Methods

**Figure 4:**
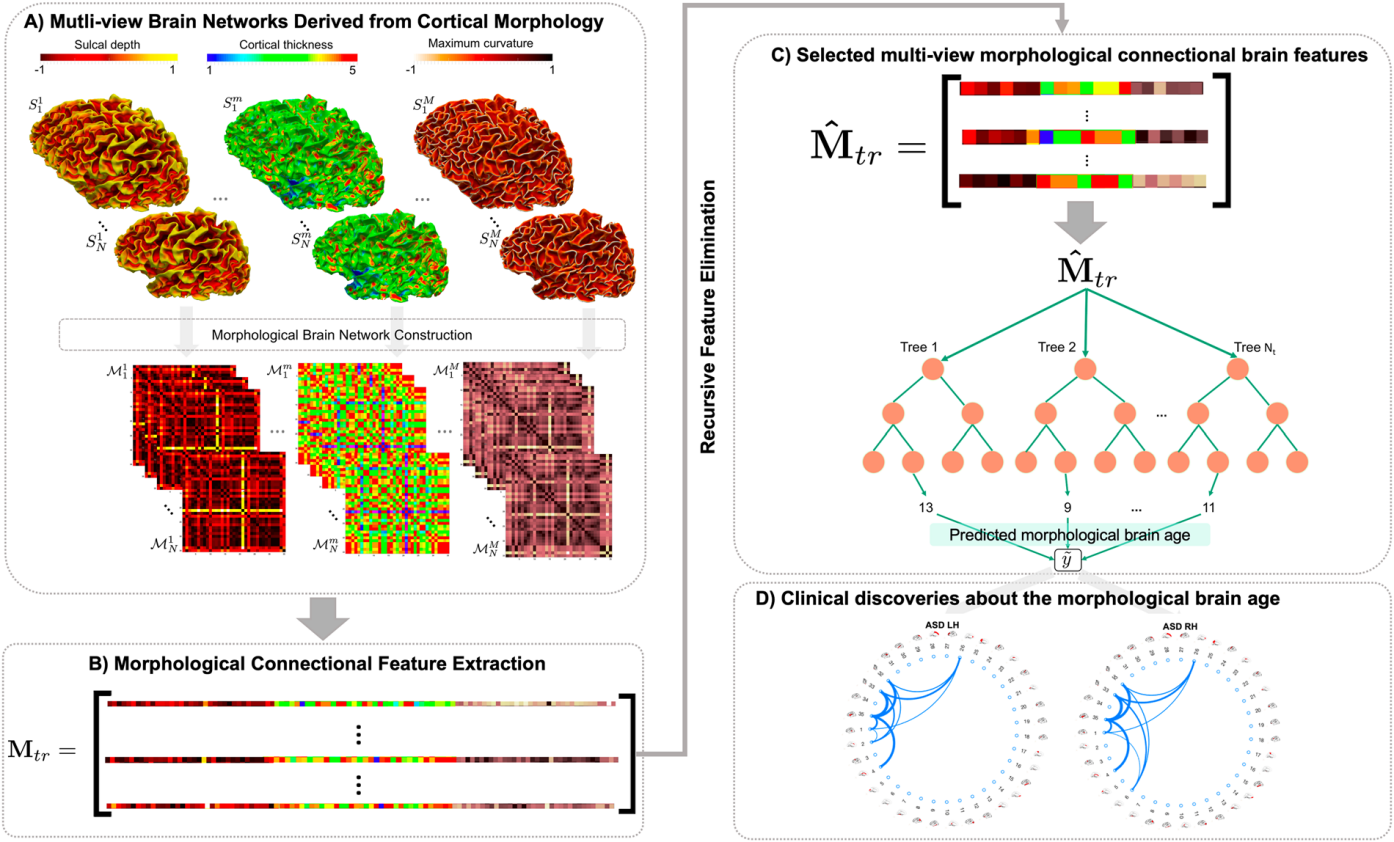
*Proposed framework for predicting morphological brain age in healthy and disordered brains*. (**A**) Construction of the multi-view brain networks from cortical morphology for each subject and the construction of the initial feature vector. For each subject $k\in \{1,\ldots ,N\}$, we derive a morphological network ${ {\mathcal M} }_{k}^{m}$ from the cortical surface ${S}_{k}^{m}$ mapped using a specific morphological attribute $m\in \{1,\ldots ,M\}$. (**B**) Next, we extract the lower triangular part of the matrix as a morphological connectional feature vector. (**C**) Reduce the dimensionality of the data and retain only the most relevant features using a feature selection method. Next, we train a Random Forest model and utilize it to predict the morphological age of a testing brain. (**D**) Connectional morphological features encoding chronological brain age.

In this section, we present the proposed framework for predicting the biological brain age using morphological brain networks. Figure 4 illustrates the four main steps of the proposed framework: (1) building multi-view morphological brain networks (M-MBN), each network capturing the dissimilarity in cortical shape between two anatomical regions using a specific cortical view (e.g., sulcal depth), (2) morphological connectional feature extraction and selection to reduce the dimensionality and remove irrelevant features, (3) training an ensemble machine-learning regression model, in this case Regression Random Forest, to predict age from M-MBN data, and (4) using the predicted morphological brain age to identify connectional morphological features that correlate highly with the chronological brain age and discover morphological brain age trends in disordered populations, specifically ASD subjects. Furthermore, to investigate the discriminative power of the predicted MBA, we used 5-fold CV to train a classifier using the disparity between the predicted MBA age and the chronological age combined with the original M-MBN data to classify disordered and healthy subjects.

### Multi-view morphological brain network (M-MBN) construction

For each cortical surface *S*_*k*_ reconstructed for the *k*^*th*^ subject in our cohort, we build a tensor $[{ {\mathcal M} }_{k}^{1},\ldots ,{ {\mathcal M} }_{k}^{M}]$ stacking *M* morphological networks (Fig. 4A). Each MBN ${ {\mathcal M} }_{k}^{m}$, of size *R* × *R* quantifies the dissimilarity in morphology between two cortical regions of interest (ROIs) using a specific cortical attribute *m* (e.g., cortical thickness). *R* denotes the number of nodes in the morphological brain network (or cortical ROIs) and *M* is the number of different cortical attributes. Each element ${ {\mathcal M} }_{k}^{m}(i,j)$ represents the absolute difference between the average value of the cortical attribute in two ROIs *i* and *j*.

Remark: In the spirit of functional brain networks that model the correlation between firing neurons and not their physical connection, our morphological brain networks model the dissimilarity in morphology between anatomical brain regions^36^. The only physical brain connectivity is traditionally quantified using diffusion MRI, from which structural brain networks are derived. Nonetheless, both functional and morphological connections mirror ‘real’ connections, as there is a relationship between brain function, morphology and structure, which requires further investigation^37^.

### Morphological connectional feature extraction and selection

For each training subject, we extract a feature vector from each MBN by taking the lower triangular part of the matrix. At this point, we propose two different strategies for combining the multi-view networks: (1) concatenation of the different views (CON), and (2) averaging the views (AVG). Firstly, we concatenate the features extracted from all MBNs into a single feature vector. Then, we define the training data M_*tr*_ (Fig. 4-B). Alternatively, we combine the views by averaging them together. This has the advantage of having a reduced dimensionality; however, can lead to a loss of complementary information and may not allow us to discover information about individual view-specific features of the brain. Next, to further reduce the dimensionality of the feature vector representing each subject as well as remove features that do not relate to the prediction of age, we propose to use Recursive Feature Elimination (RFE)^38–40^ to rank and select the most relevant features. Specifically, RFE is a supervised wrapper feature selection method, which evaluates different combinations of features then ranks their predictive accuracy. Next, the ranks of these combinations are used to eliminate a subset of the features ranked the lowest. This process is repeated recursively using smaller and smaller amounts of features until all features are ranked. Ultimately, RFE returns a complete ranked vector of the features across all training subjects ${\hat{{\bf{M}}}}_{tr}$ (Fig. 4), which can then be used to train our regression model. Additionally, the feature selection process identifies features that are consistently ranked highly across training folds and can then be used to discover features which highly correlate with the MBA.

### Training a machine-learning regression model to predict morphological age from M-MBN data

Next, we train a regression random forest to predict a subject’s age from the M-MBN (Fig. 4C). Regression Random Forest is an ensemble regression method that works by creating many decision trees. Each decision tree is then trained using a different random subset of subjects from the initial training data for each tree. Once each tree has completed and has a prediction value then results are aggregated by calculating the mean of all the trees’ prediction values. This mean value is then returned as the predicted age. To learn the model, we divide the data into training and testing sets. This is achieved in two ways. *First*, a first model is learned *individually* for healthy and disordered populations. *Secondly*, a second model is trained using *only* healthy brains to produce a ‘baseline healthy model’. Then, we test the learned healthy model on disordered subjects to predict their morphological brain age. The absolute difference between the predicted morphological age and the chronological age for disordered brains can be potentially used as a disorder biomarker^41^.

### Identification of morphological connectional features fingerprinting brain age

The final step in the proposed framework is three-fold. First, we investigate if certain morphological features are more affected by age in disordered brains compared with healthy brains. Specifically, we aim to identify connectional morphological features which are most linked to biological age. To do so, we define a relevance score for each connectional morphological feature by averaging its rank across different folds of cross-validation. Next, we identify the top *K* features associated with morphological brain age.

We would like to note that all methods and experimental protocols were carried out using the public Autism Brain Imaging Data Exchange (ABIDE) dataset. Informed consent was obtained from all ABIDE subjects or, if subjects are under 18, from a parent and/or legal guardian.

## Supplementary information

Supplementary Table

## Data Availability

The data that support the findings of this study are available from ADBIDE data (http://fcon_1000.projects.nitrc.org/indi/abide/). The Matlab code is also available from the authors upon request.
