# Supplementary material for: Morphological Brain Age Prediction using Multi-View Brain Networks Derived from Cortical Morphology in Healthy and Disordered Participants
Source: Sci Rep. 2019 Jul 4;9:9676. doi: 10.1038/s41598-019-46145-4 (PMC6609705; doi:10.1038/s41598-019-46145-4)
Supplement: Supplementary file 1 — Supplementary Table [file 41598_2019_46145_MOESM1_ESM.docx]

**Morphological Brain Age Prediction using Multi-View Brain Networks Derived from Cortical Morphology in Healthy and Disordered Participants**

Joshua Corps^1^ and Islem Rekik^1,2*^

^1^BASIRA lab, CVIP group, School of Science and Engineering, Computing, University of Dundee, Dundee, UK

^2^Faculty of Computer and Informatics, Istanbul Technical University, Istanbul, Turkey

Correspondence and requests for materials should be addressed to I. Rekik ([irekik@dundee.ac.uk](mailto:irekik@dundee.ac.uk))

Address: Queen Mother Building

University of Dundee

BASIRA Lab ([www.basira-lab.com](http://www.basira-lab.com))

DD1 4HN Scotland, UK

Office: Office 2.18 QMB

Tel: +44 (0)1382 384908

Email: irekik@dundee.ac.uk

**Table 1.** Data distribution of the ABIDE dataset used for testing.

|  | NC | ASD |
| --- | --- | --- |
| Male | 140 | 155 |
| Female | 15 | 31 |
| Total | 155 | 186 |
| Mean age | 16.92 | 16.65 |
| Std age | 6.38 | 6.06 |

**Table 2.** Comparison of age prediction using **ASD RH** data with 5-fold cross-validation by our method and comparison methods. R is the correlation between the predicted ages and the ground truth ages, and P is the p-value of their statistical difference (E denotes exponent). MAE denotes the mean absolute error between the predicted ages and the ground truth ages.

| Method | Dataset | R | P | MAE |
| --- | --- | --- | --- | --- |
| CPM | View 1 | 0.43 | 3.09E-08 | 4.25 |
|  | View 2 | 0.72 | 1.77E-26 | 3.39 |
|  | View 3 | 0.45 | 2.89E-09 | 4.18 |
|  | View 4 | 0.44 | 1.25E-08 | 4.45 |
|  | Averaged Views | 0.73 | 7.60E-27 | 3.33 |
|  |  |  |  |  |
| SVR | View 1 | 0.41 | 8.20E-08 | 4.15 |
|  | View 2 | 0.63 | 1.36E-18 | 4.32 |
|  | View 3 | 0.38 | 9.28E-07 | 4.24 |
|  | View 4 | 0.05 | 5.21E-01 | 4.64 |
|  | Averaged Views | 0.72 | 3.51E-26 | 3.28 |
|  | Concatenated Views | 0.62 | 4.65E-18 | 4.55 |
|  |  |  |  |  |
| SVR+RFE | View 1 | 0.42 | 6.28E-08 | 4.15 |
|  | View 2 | 0.69 | 7.92E-23 | 3.43 |
|  | View 3 | 0.43 | 2.33E-08 | 4.16 |
|  | View 4 | 0.05 | 5.21E-01 | 4.64 |
|  | Averaged Views | 0.73 | 6.97E-27 | 3.24 |
|  | Concatenated Views | 0.70 | 9.16E-24 | 3.49 |
|  |  |  |  |  |
| RF | View 1 | 0.56 | 3.26E-14 | 3.97 |
|  | View 2 | 0.71 | 3.09E-25 | 3.51 |
|  | View 3 | 0.41 | 1.29E-07 | 4.34 |
|  | View 4 | 0.42 | 3.93E-08 | 4.44 |
|  | Averaged Views | 0.69 | 1.41E-23 | 3.57 |
|  | Concatenated Views | 0.72 | 1.50E-26 | 3.55 |
|  |  |  |  |  |
| RF+RFE  (ours) | View 1 | 0.58 | 2.40E-15 | 3.88 |
|  | View 2 | 0.72 | 7.51E-26 | 3.43 |
|  | View 3 | 0.46 | 2.57E-09 | 4.13 |
|  | View 4 | 0.41 | 1.30E-07 | 4.49 |
|  | Averaged Views | 0.72 | 5.22E-26 | 3.47 |
|  | Concatenated Views | **0.76** | **4.14E-30** | **3.38** |

**Table 3.** Comparison of age prediction using **NC RH** data with 5-fold cross-validation by our method and comparison methods. R is the correlation between the predicted ages and the ground truth ages, and P is the p-value of their statistical difference (E denotes exponent). MAE denotes the mean absolute error between the predicted ages and the ground truth ages.

| Method | Dataset | R | P | MAE |
| --- | --- | --- | --- | --- |
| CPM | View 1 | 0.40 | 1.75E-08 | 4.15 |
|  | View 2 | 0.63 | 5.91E-22 | 3.52 |
|  | View 3 | 0.38 | 1.14E-07 | 4.24 |
|  | View 4 | 0.34 | 2.62E-06 | 4.30 |
|  | Averaged Views | 0.65 | 2.42E-23 | 3.55 |
|  |  |  |  |  |
| SVR | View 1 | 0.33 | 5.42E-06 | 4.10 |
|  | View 2 | 0.66 | 7.91E-25 | 3.53 |
|  | View 3 | 0.31 | 1.29E-05 | 4.31 |
|  | View 4 | 0.07 | 3.20E-01 | 4.33 |
|  | Averaged Views | 0.71 | 2.69E-29 | 3.18 |
|  | Concatenated Views | 0.70 | 9.61E-29 | 3.37 |
|  |  |  |  |  |
| SVR+RFE | View 1 | 0.33 | 4.04E-06 | 4.10 |
|  | View 2 | 0.66 | 7.91E-25 | 3.53 |
|  | View 3 | 0.33 | 5.48E-06 | 4.16 |
|  | View 4 | 0.07 | 3.18E-01 | 4.33 |
|  | Averaged Views | 0.71 | 2.69E-29 | 3.18 |
|  | Concatenated Views | 0.70 | 9.50E-29 | 3.37 |
|  |  |  |  |  |
| RF | View 1 | 0.52 | 1.95E-14 | 3.88 |
|  | View 2 | 0.68 | 2.86E-26 | 3.26 |
|  | View 3 | 0.42 | 2.20E-09 | 4.07 |
|  | View 4 | 0.27 | 1.48E-04 | 4.37 |
|  | Averaged Views | 0.72 | 2.20E-31 | 3.12 |
|  | Concatenated Views | 0.68 | 1.04E-26 | 3.34 |
|  |  |  |  |  |
| RF+RFE  (ours) | View 1 | 0.54 | 2.42E-15 | 3.84 |
|  | View 2 | 0.72 | 2.26E-31 | 3.09 |
|  | View 3 | 0.44 | 3.64E-10 | 4.04 |
|  | View 4 | 0.31 | 1.97E-05 | 4.38 |
|  | Averaged Views | 0.73 | 8.71E-32 | 3.04 |
|  | Concatenated Views | **0.73** | **5.91E-32** | **3.03** |
